# Miniaturised ultrasound evaluation at the bedside

**DOI:** 10.1007/s00345-022-04018-y

**Published:** 2022-05-18

**Authors:** Katharina Hollerieth, Minh-Truc Vo-Cong, Stephanie Preuss, Stephan Kemmner, Konrad Friedrich Stock

**Affiliations:** 1 Department of Nephrology, School of Medicine, Technical University of Munich, Munich, Germany; 2 Present Address: Transplant Centre, University Hospital Munich, Ludwig-Maximilians-University (LMU), Munich, Germany

**Keywords:** Point-of-care systems, Ultrasonography, Diagnostic imaging, Kidney, Nephrology, Urology

## Abstract

**Purpose:**

“Point-of-Care Ultrasound” (POCUS) is now a familiar term. Although the European Federation of Societies for Ultrasound in Medicine and Biology (ESFUMB) published a position paper about its usage (Nielsen et al. in Ultraschall Med 40(1):30–39. 10.1055/a-0783-2303, 2019), there has not been much scientific focus on its utility in uro-nephrological clinical practice thus far. The aim of this study was to evaluate the present usage of pocket ultrasound devices at the bedside.

**Methods:**

27 investigators (all medical doctors with at least 6 months of experience in sonography) performed 280 bedside examinations using a pocket ultrasound device for common clinical issues.

**Results:**

The most frequent indications included evaluation of hydronephrosis (147), volume management including assessment of dimension of the vena cava inferior (IVC) (195), detection of pleural, pericardial and abdominal effusions (113) as well as residual urine (52). In 90%, specific clinical questions were effectively answered by the pocket ultrasound device alone.

**Conclusions:**

POCUS can be useful in the uro-nephrological field. In the hands of an experienced investigator, it saves time and, when it is realised that departmental ultrasound is not cheap, there is also an economic benefit with applicability within both inpatient and outpatient clinic settings. While acknowledging its technical limits, pocket ultrasound devices may nevertheless be helpful in targeted situations for triage or for bedside follow-up exams after earlier high-end ultrasound-based diagnosis.

**Supplementary Information:**

The online version contains supplementary material available at 10.1007/s00345-022-04018-y.

## Introduction

“Point-of-Care Ultrasound” is a familiar term, with its acronym ‘POCUS’ posing quite the popular rejoinder, which describes a real-time ultrasonic bedside examination focussed on addressing specific clinical questions allowing immediate interpretation by the examining physician [1]. Improving the diagnostic utility within physical examination, it heuristically speeds interpretation of findings towards advancing clinical diagnostic reasoning and guiding therapy.

Technological advances have enabled such miniaturised hand-held devices to widen the POCUS concept. Former studies investigated their use in different fields including abdominal [2–4] and cardiac [5] as well as in other settings including clinical rounds [6], intensive care medicine [7, 8] and pre-hospital emergency medicine [9].

POCUS invites interest within Uro-Nephrology [10], but there has been little scientific scrutiny in evaluating its utility within this craft. Ultrasound imaging is an important diagnostic contribution to differentiating between pre-, intra- and postrenal causes of kidney failure as well as acute versus chronic kidney insufficiency. Furthermore, when integrated within the physical examination, it effects surrogate evaluation of IVC to reflect the patient´s volume status. As many patients are immobile because of sickness or age, a focussed bedside sonographic examination using hand-held ultrasound devices (HHUD) may be helpful and time saving where appropriately indicated. However, evaluating this tool’s utility in immediate retrospect at the end of an evaluation is an important component, reflective, skill to acquire towards safe practice. Limitations inherent to miniaturisation may compromise image quality, subsequently reducing diagnostic accuracy [3]. But nonetheless, POCUS might accept some degree of loss of accuracy to the benefit of the possibility of its timely, direct and bedside usage in the hands of the trained physician. Of course, that component skill should be emphasised and should be the basis of any comparison.

In this study the present role of HHUD used bedside for the daily clinical routine of Uro-Nephrology was evaluated.

## Methods

**Fig. 1:**
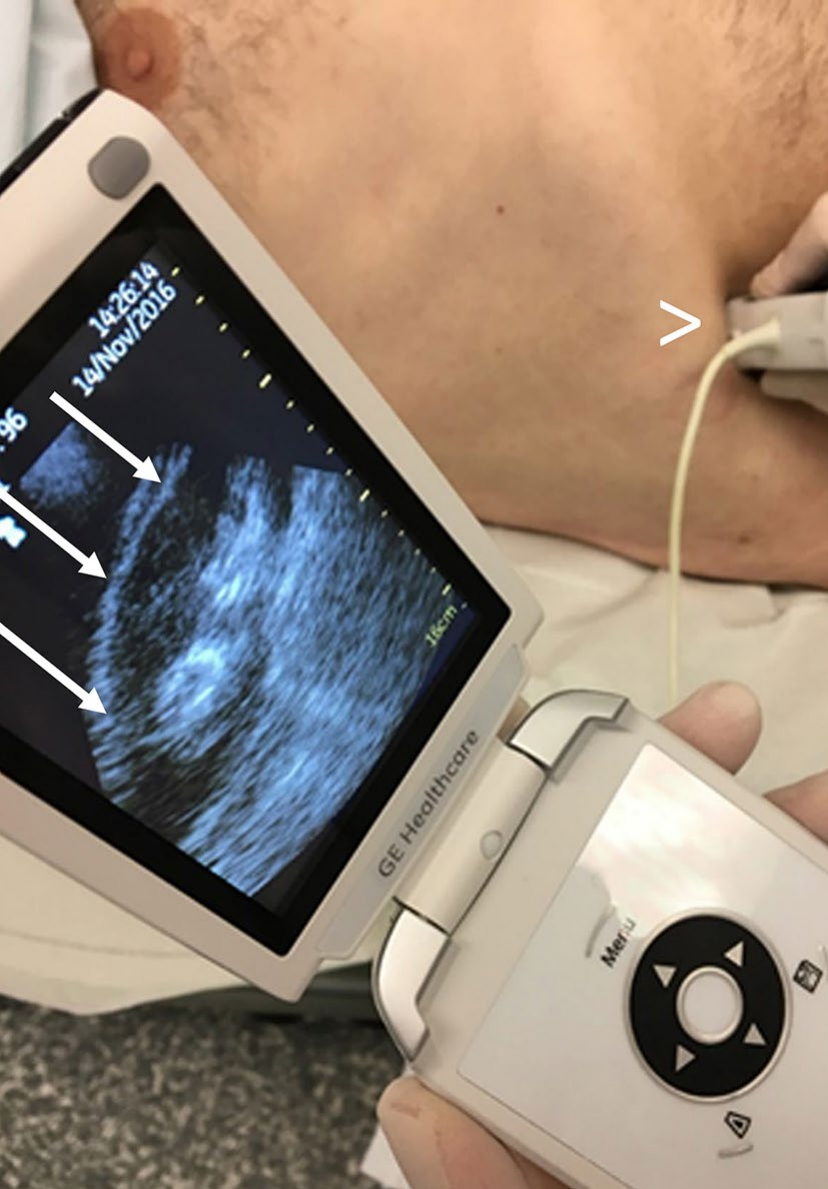
Examination with the HHUD GE Vscan v1.2: the probe (>) is positioned in the upper abdomen of the patient. On the monitor of the HHUD you can see the right kidney (→)

All examinations were performed with the GE Vscan v1.2 (GE Medical Systems, Milwaukee, WI, USA; broad bandwidth phased array transducer: 1.7–3.8 MHz; Fig. 1). Stationed in our uro-nephrological ultrasound department, the HHUD could be used whenever needed for sonographic evaluation of patients on our nephrology/ rheumatology ward, for patients while on dialysis, in the nephrological outpatient clinic or for any other nephrological consultation in the hospital. The investigators were all ultrasound-experienced physicians in our nephrology department, who had all undertaken full-time sonographic training for a minimum of 6 months and had received a detailed introduction in the use of HHUD.

After each HHUD examination, the investigators were encouraged to fill a questionnaire including age and gender of the patient, indication for the examination and whether they considered the issues being evaluated could be answered by HHUD, or if another examination by a high-end ultrasound system (HEUS) was necessary.

This observational study was approved by the ethics committee of the Technical University of Munich and conducted in accordance with its guidelines.

For data management and evaluation Excel (Microsoft Office 365 Pro Plus; Microsoft Corp., Redmond, Washington, USA) was used.

## Results

**Fig. 2:**
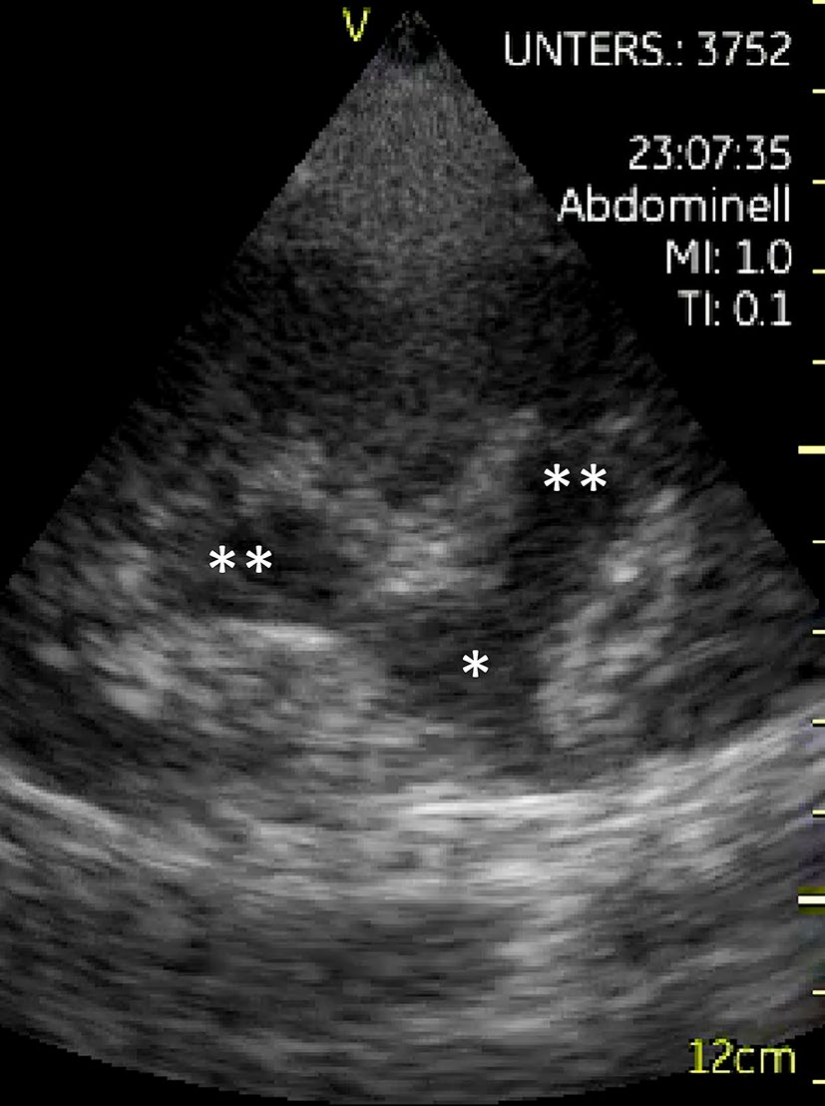
Hydronephrosis °II, diagnosed with the HHUD GE Vscan v1.2: dilatation of the renal pelvis (*) and the renal calices (**)

**Fig. 3:**
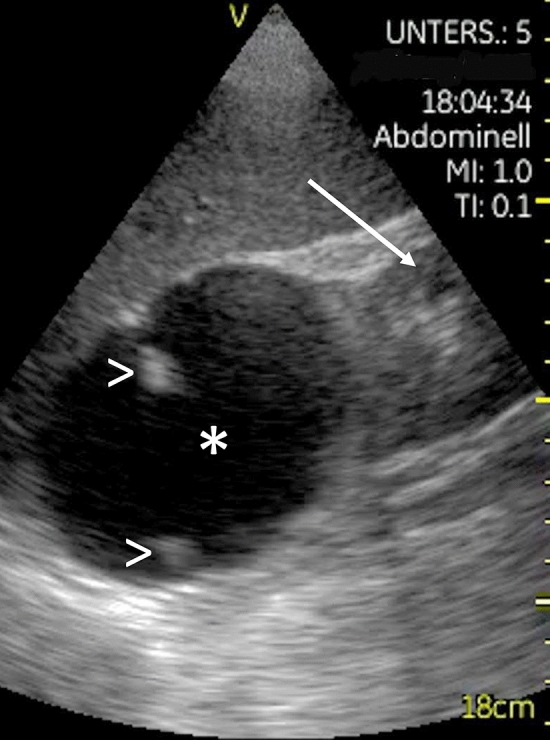
Cyst (*) in the upper pole of the right kidney (→), depicted with the HHUD GE Vscan v1.2, with a partially calcified septum (>)

From January 21, 2016, to January 17, 2018, 280 ultrasound examinations were performed by 27 different investigators. On average, the patients were 68.1 years old (178 males and 102 females). The HHUD was used for the following indications (number in brackets; online resource 1): evaluation of hydronephrosis (147; Fig. 2), evaluation of the vena cava inferior (195: online resource 2 a and b), revealing effusions (113; pleural: online resource 3, intraabdominal: online resource 4, pericardial), measuring residual urine in the bladder (52) and for other indications (18), including imaging of the thyroid (1), the neck (1), the joints (2), the abdominal aorta (2; online resource 5 a and b), the heart (3), the kidney (3 times for control of bleeding after renal biopsy; once for evaluation of kidney cysts: Fig. 3) and the intestines (1; because of lower abdominal pain). Moreover, it was used three times for ultrasonic guided puncture of pleural effusion and once to guide knee aspiration.

In seven examinations (2.5%), a precise sonographic evaluation by the HHUD was not possible and the examination had to be repeated with HEUS in the following cases (see Table 1): evaluation of a joint (1), aspiration of a knee (1), sonography of the aortic valve (1), sonography of the neck (1), imaging of the kidney (3): once for confirming hydronephrosis, once for imaging the left kidney in an adipose patient and once in a patient with lower abdominal pain (with HEUS, bleeding into a renal cyst could be detected).

**Table 1:** List of cases where HHUD failed: an evaluation with HHUD was not possible in these cases

Cases where pocket ultrasound device failed	Number
Joints	2
Heart	1
Kidney	
- Exclusion of hydronephrosis	1
- Imaging left kidney	1
- Haemorrhagic renal cyst	1
Cervical lymph nodes	1

21 examinations (7.5%) were followed by a HEUS as not all issues could be answered by the HHUD alone: in these cases, the examination with HHUD at least revealed a problem that had to be evaluated by HEUS (see Table 2). For example, suspicion of kidney stone had to be confirmed by HEUS and other methods (CT-KUB). In two other patients hydronephrosis was detected: HEUS could differentiate a dilated calyceal system from one or many renal cortical/parapelvic cysts and allow an accurate grading of hydronephrosis due to the higher resolution and also 3-D-option of the ultrasound device. A diverticulum of the urinary bladder and clots of blood in the urinary bladder (online resource 6), kidney cysts (Fig. 3) and macro vascularization of the (transplanted) kidney were other examples requiring further evaluation by HEUS in this study.

**Table 2:** List of cases, where examination by HHUD was followed by a HEUS as not all questions could be answered

Further evaluation by high-end ultrasound machine	Number	Comment
Kidney		
- Stones	2	
- parenchymal/cysts	3	
- RI-values	3	
- Grading of hydronephrosis	2	
- Transplant kidney vascularisation	1	
Extent of pleural effusion	1	Reassurance in adipose patient
Intestines	1	
Diverticulum of the urinary bladder	1	
Blood clots in the urinary bladder	1	
Knee: extent of effusion	1	
H. o. thyreoidectomy	1	Exclusion of residual thyroidal tissue
Low battery	1	
Puncture of pleural effusion	1	Bad examination conditions
V. cava inferior	1	Reassurance in adipose patient
Doppler of the groin	1	

## Discussion

In 90% of all examinations, HHUD revealed satisfactory results in accordance with other studies proving its feasibility in addressing specific clinical questions [11, 12].

In only seven of 280 examinations, HHUD provided no benefit: this may be due in part by bad examination conditions, which are more difficult to handle with an HHUD than with an HEUS (small monitor and limited technical specification), and/or by inappropriate indications. For example, a standard quality evaluation of the neck or the joints is not achievable with a small device without a high frequency transducer. Such attempts were especially observed in the initial period of using HHUD and the investigators quickly became familiar with the limitations of HHUD.

In 21 of 280 examinations, further HEUS examination was needed for more detailed evaluation. In many of these cases HEUS was akin to verification: the pathology was already suspected after HHUD but required to be confirmed by HEUS. In some cases, HHUD was technically limited by constitutional factors of the patients e.g., high BMI.

Previous HHUD studies required that the investigators were possessed of adequate experience to achieve satisfactory results [13, 14], and standardized training curricula and examination protocols were a pre-requisite for the individuals performing the examinations: POCUS-examinations by HHUD might be faster to perform and limited in their scope but are not easier. In our study, only physicians experienced in abdominal sonography for at least six months used the HHUD.

The patient´s average age in this study (68.1 years) was representative of the uro-nephrological patient population which typically is comprised of an older profile which may reflect a coincidence of higher age and immobility: it is often easier and faster to perform a bedside consultation with HHUD instead of bringing an immobilized patient to the HEUS. Other features of a shorter boot-up time, supplanted transfer and an easier mobility for positioning at the patient´s bedside, predicate that it is easier to insert an HHUD exam in the patient’s care journey, than a formal requesting of HEUS with its inherent delays [3].

A limitation of this study is the lack of a comparison to gold standard examination—that would be (at least) an HEUS examination. As there was no control of the quality of the examinations, investigators might have been lulled into a false sense of security and therefore did not initiate further investigations. To our knowledge, no relevant finding or diagnosis was missed by HHUS concerning the clinical issue. Moreover, the feasibility of abdominal ultrasound for specific issues by HHUD was already shown in other studies [2–4, 11, 12, 15–17].

It is further an acknowledged limitation that not all HHUD examinations were formally documented by the investigators, using the above-mentioned questionnaire, due to limited time in daily clinical routine. This might have biased our results to a certain degree. However, within an observational period of almost two years the presented results seemed by consensus to be representative. Moreover, we included different settings (ward, dialysis, in- and outpatient clinics), covering a broad spectrum of uro-nephrological patients and practice.

As described in an article by Osterwalder, traditional comprehensive ultrasound and POCUS complement rather than compete with one another: the HHUD is not meant to replace the HEUS or the complete ultrasound examination—it has its applications parallel to those [18]. Our study design considered that the physicians still retained the option to request a departmental ultrasound in the form of HEUS performed by a full-time ultrasound consultant, without performing any HHUD beforehand. On the one hand, this implies a selection bias regarding the success rate: as the clinicians selected those for HHUD where they thought it was likely to yield an answer, “difficult cases” might be underrepresented, making the success rate higher than it actually is. On the other hand, this better reflects the clinical reality: clinicians should always use those diagnostic methods they suspect best success of.

Regarding the results of our study, HHUD incorporated into uro-nephrological clinical practice is especially useful for a rapid evaluation of circulating volume status including the v. cava inf. (online resource 2 a and b) and pleural/intraabdominal (online resource 3 and 4) or pericardial effusions, for evaluation of the kidneys (size, renal parenchyma, exclusion of hydronephrosis, gross vascularisation confirmation by Colour Doppler ultrasound respecting the limited options; online resource 7 and Fig. 2) and the urinary bladder (residual urine). If solid organ pathologies are suspected or the precise evaluation of vascularisation (e.g., including RI-values) is needed, HEUS is indicated.

Meanwhile, there are pocket ultrasound devices equipped with a convex and a linear scanner. Moreover, additional modes like Pulsed-wave Doppler are becoming available: hereby new areas of applications may arise such as ultrasound-guided cannulation in haemodialysis vascular access (online resource 8). [19]

## Conclusion

To summarize, POCUS by HHUD can be incorporated into clinical uro-nephrological practice. It is a helpful tool for rapid exclusion of hydronephrosis, evaluation of circulatory blood volume status (IVC and effusions) and measurement of the residual volume of the urinary bladder. However, investigators should be aware of its limitations, e.g., in the assessment of solid organ pathologies, such as parenchymal disease and space-occupying lesions, as well as in adverse examination conditions (e.g., very raised BMI). There is ongoing technical development of HHUD with high frequency arrays and further ultrasound modes which might even widen the spectrum of indications for HHUD; further studies are needed to evaluate their applicability.

## Supplementary Information

Below is the link to the electronic supplementary material.

Online Resource 1 Indications for the usage of the HHUD in our uro-nephrological ultrasound department: in several patients, there was more than one indication (JPG 78 KB)

Online Resource 2 a and b V. cava inferior (IVC) (→) – two examples (a narrow IVC, b wide IVC) (TIF 12287 KB)

Supplementary file3 (TIF 12326 KB)

Online Resource 3 Pleural effusion (*) (TIF 6921 KB)

Online Resource 4 Ascites (→), surrounding the liver (*) (TIF 12326 KB)

Online Resource 5 a and b Abdominal aorta with aneurysm (*) (TIF 12326 KB)

Supplementary file7 (TIF 12330 KB)

Online Resource 6 Blood clot (→) in the urinary bladder (*) (TIF 12460 KB)

Online Resource 7 Kidney: measurement of the renal size (TIF 2699 KB)

Online Resource 8 Cannulation of a haemodialysis fistula: on the monitor of the HHUD you can see the needle (→) placed in the haemodialysis fistula (*) (TIF 10746 KB)

## References

[1] Nielsen MB, Cantisani V, Sidhu PS, Badea R (2019). The use of handheld ultrasound devices—an EFSUMB position paper. Ultraschall Med.

[2] Tse KH, Luk WH, Lam MC (2014). Pocket-sized versus standard ultrasound machines in abdominal imaging. Singap Med J.

[3] Stock KF, Klein B, Steubl D, Lersch C (2015). Comparison of a pocket-size ultrasound device with a premium ultrasound machine: diagnostic value and time required in bedside ultrasound examination. Abdom Imaging.

[4] Barreiros AP, Cui XW, Ignee A, De Molo C (2014). EchoScopy in scanning abdominal diseases: initial clinical experience. Z Gastroenterol.

[5] Mirabel M, Celermajer D, Beraud AS, Jouven X (2015). Pocket-sized focused cardiac ultrasound: strengths and limitations. Arch Cardiovasc Dis.

[6] Khan HA, Wineinger NE, Uddin PQ, Mehta HS (2014). Can hospital rounds with pocket ultrasound by cardiologists reduce standard echocardiography?. Am J Med.

[7] Melamed R, Sprenkle MD, Ulstad VK, Herzog CA (2009). Assessment of left ventricular function by intensivists using hand-held echocardiography. Chest.

[8] Biais M, Carrie C, Delaunay F, Morel N (2012). Evaluation of a new pocket echoscopic device for focused cardiac ultrasonography in an emergency setting. Crit Care.

[9] Gustafsson M, Alehagen U, Johansson P (2015). Imaging congestion with a pocket ultrasound device: prognostic implications in patients with chronic heart failure. J Card Fail.

[10] Niyyar VD, O'Neill WC (2018). Point-of-care ultrasound in the practice of nephrology. Kidney Int.

[11] Barreiros AP, Dong Y, Ignee A, Wastl D (2019). EchoScopy in scanning abdominal diseases; a prospective single center study. Med Ultrason.

[12] Colli A, Prati D, Fraquelli M, Segato S (2015). The use of a pocket-sized ultrasound device improves physical examination: results of an in- and outpatient cohort study. PLoS ONE.

[13] Ojeda JC, Colbert JA, Lin X, McMahon GT (2015). Pocket-sized ultrasound as an aid to physical diagnosis for internal medicine residents: a randomized trial. J Gen Intern Med.

[14] Charron C, Templier F, Goddet NS, Baer M (2015). Difficulties encountered by physicians in interpreting focused echocardiography using a pocket ultrasound machine in prehospital emergencies. Eur J Emerg Med.

[15] Lavi A, Tzemah S, Hussein A, Bishara I (2017). A urologic stethoscope? Urologist performed sonography using a pocket-size ultrasound device in the point-of-care setting. Int Urol Nephrol.

[16] Kameda T, Uebayashi K, Wagai K, Kawai F (2001). (2018) Assessment of the renal collecting system using a pocket-sized ultrasound device. J Med Ultrason.

[17] Fröhlich E, Beller K, Muller R, Herrmann M (2020). Point of care ultrasound in geriatric patients: prospective evaluation of a portable handheld ultrasound device. Ultraschall Med.

[18] Osterwalder J, Tercanli S (2018). POCUS—chance or risk?. Ultraschall Med.

[19] Schoch ML, Currey J, Orellana L, Bennett PN (2018). Point-of-care ultrasound-guided cannulation versus standard cannulation in haemodialysis vascular access: protocol for a controlled random order crossover pilot and feasibility study. Pilot Feasibility Stud.

