# Supplementary figures and images for: Miniaturised ultrasound evaluation at the bedside
Source: World J Urol. 2022 May 18;41(3):635–40. doi: 10.1007/s00345-022-04018-y (PMC10082701; doi:10.1007/s00345-022-04018-y)

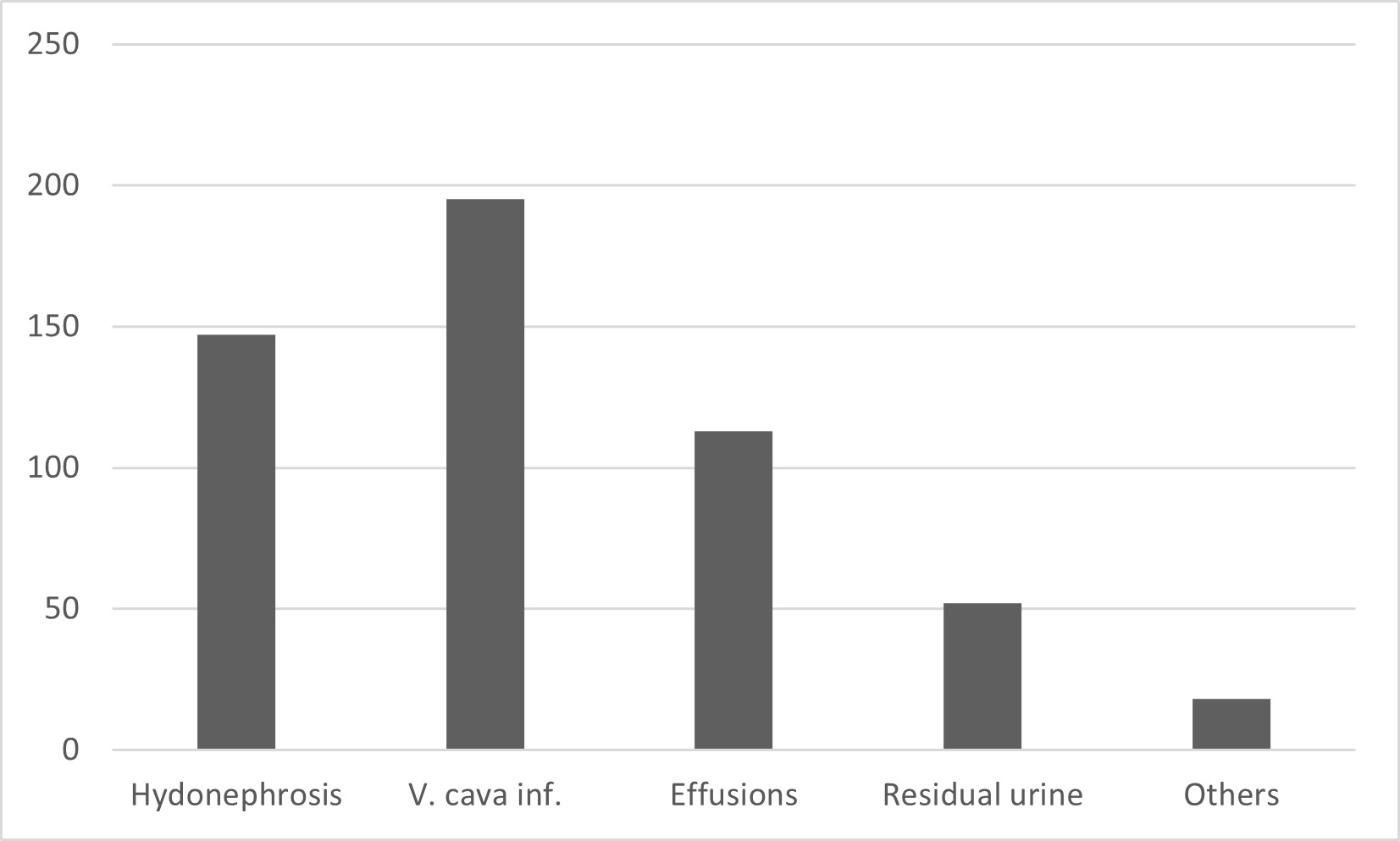

Supplement: Supplementary file 1 — Online Resource 1 Indications for the usage of the HHUD in our uro-nephrological ultrasound department: in several patients, there was more than one indication (JPG 78 KB) [file 345_2022_4018_MOESM1_ESM.jpg]

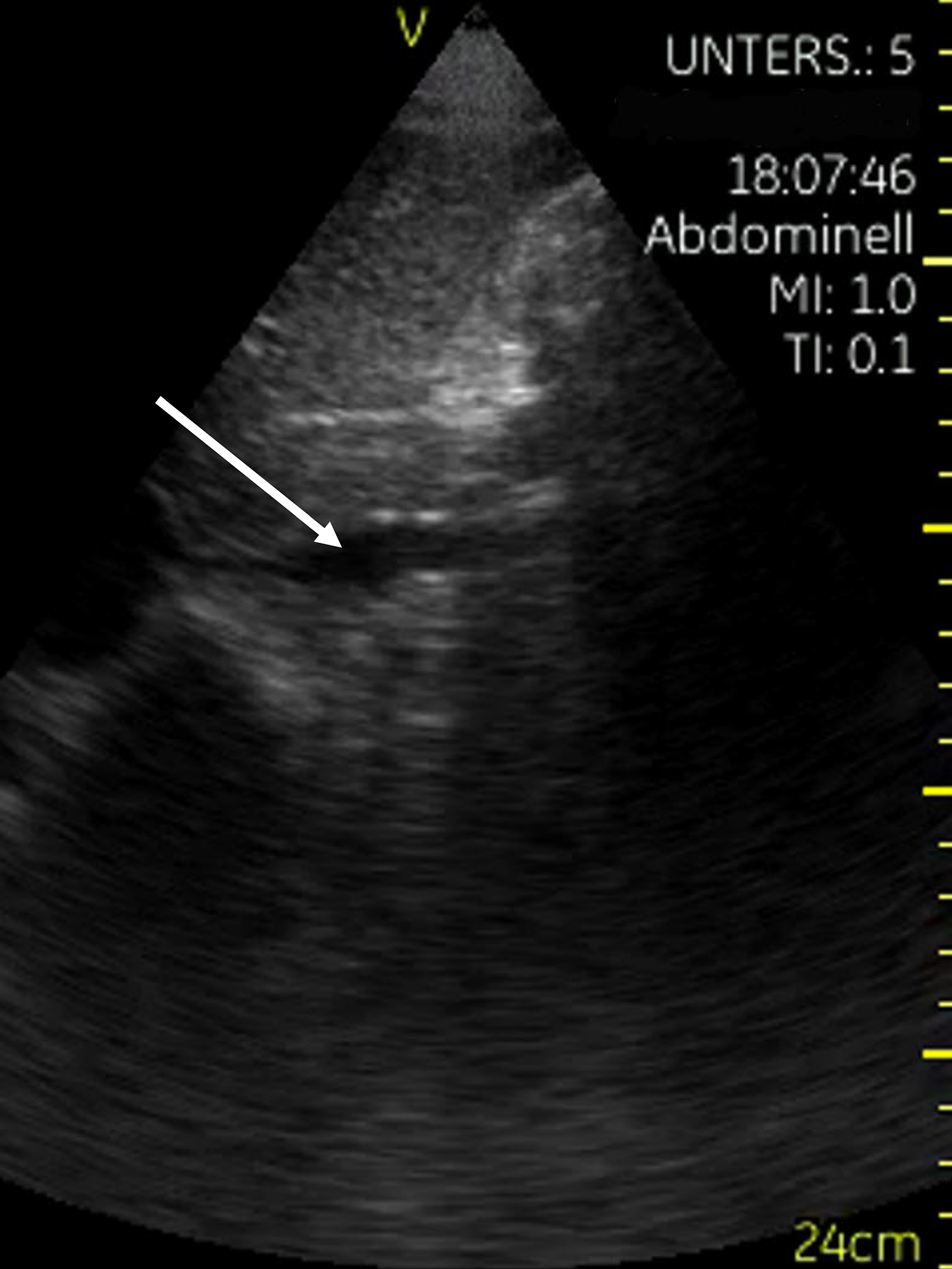

Supplement: Supplementary file 2 — Online Resource 2 a and b V. cava inferior (IVC) (→) – two examples (a narrow IVC, b wide IVC) (TIF 12287 KB) [file 345_2022_4018_MOESM2_ESM.tif]

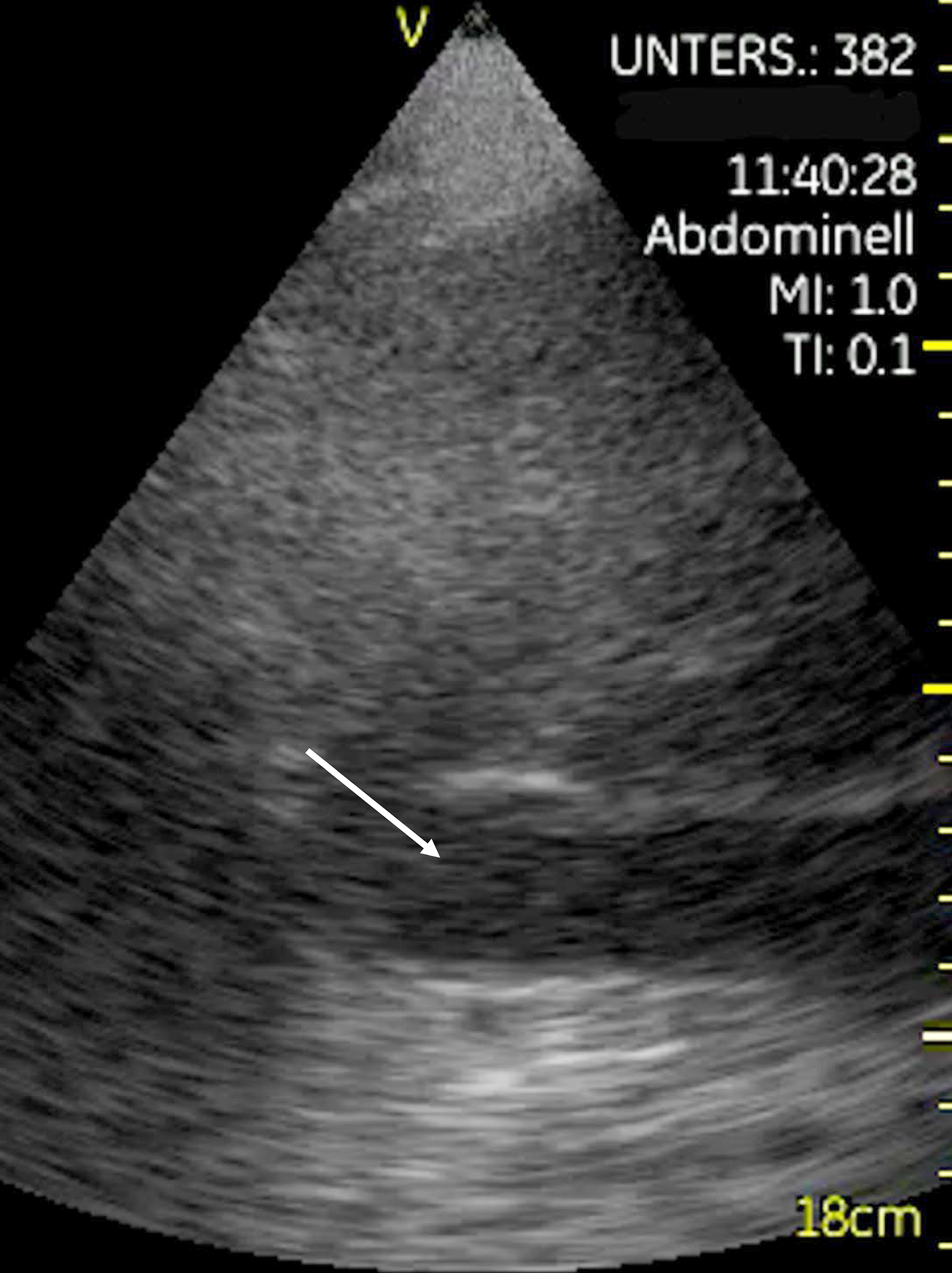

Supplement: Supplementary file 3 — Supplementary file3 (TIF 12326 KB) [file 345_2022_4018_MOESM3_ESM.tif]

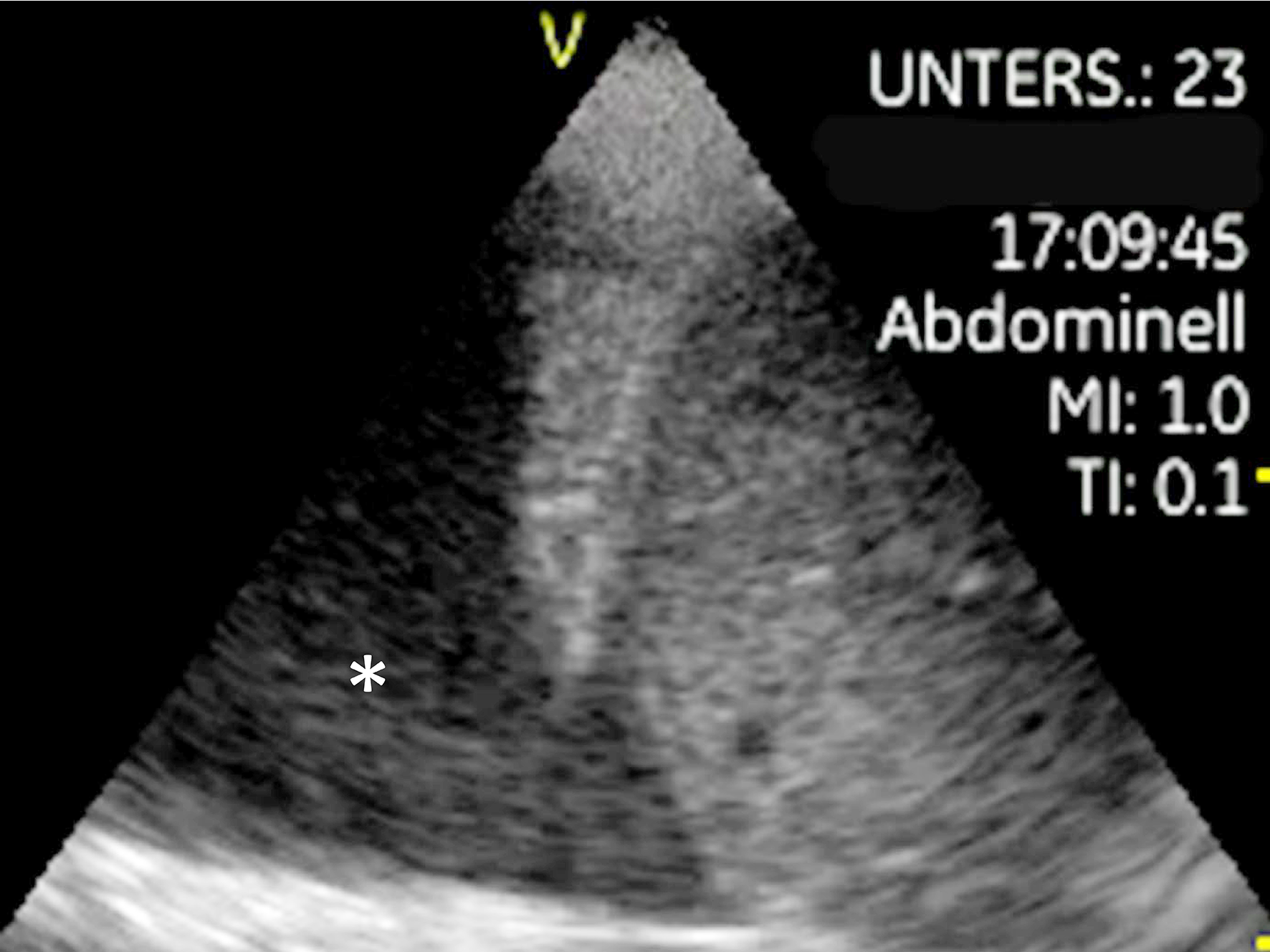

Supplement: Supplementary file 4 — Online Resource 3 Pleural effusion (*) (TIF 6921 KB) [file 345_2022_4018_MOESM4_ESM.tif]

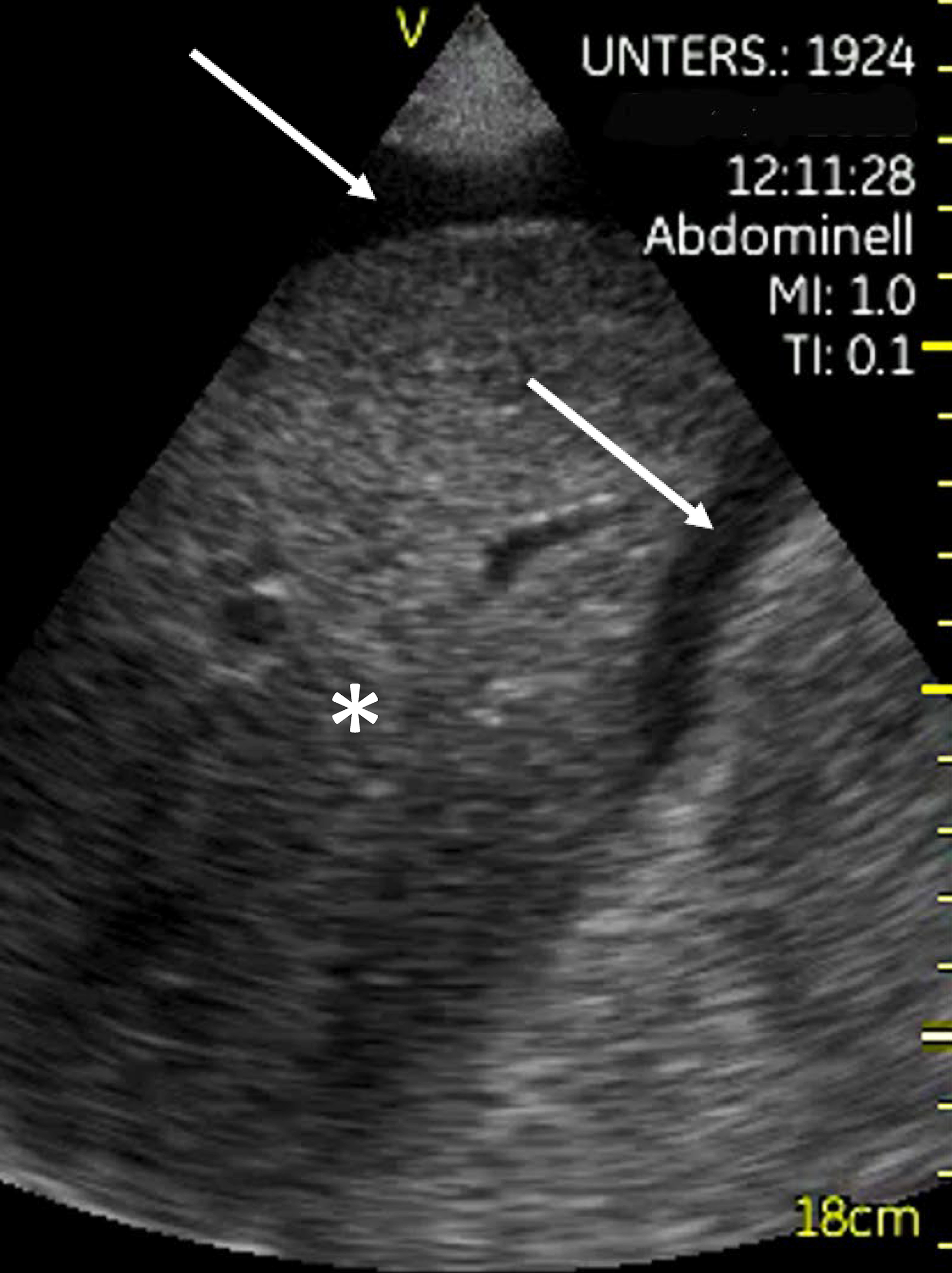

Supplement: Supplementary file 5 — Online Resource 4 Ascites (→), surrounding the liver (*) (TIF 12326 KB) [file 345_2022_4018_MOESM5_ESM.tif]

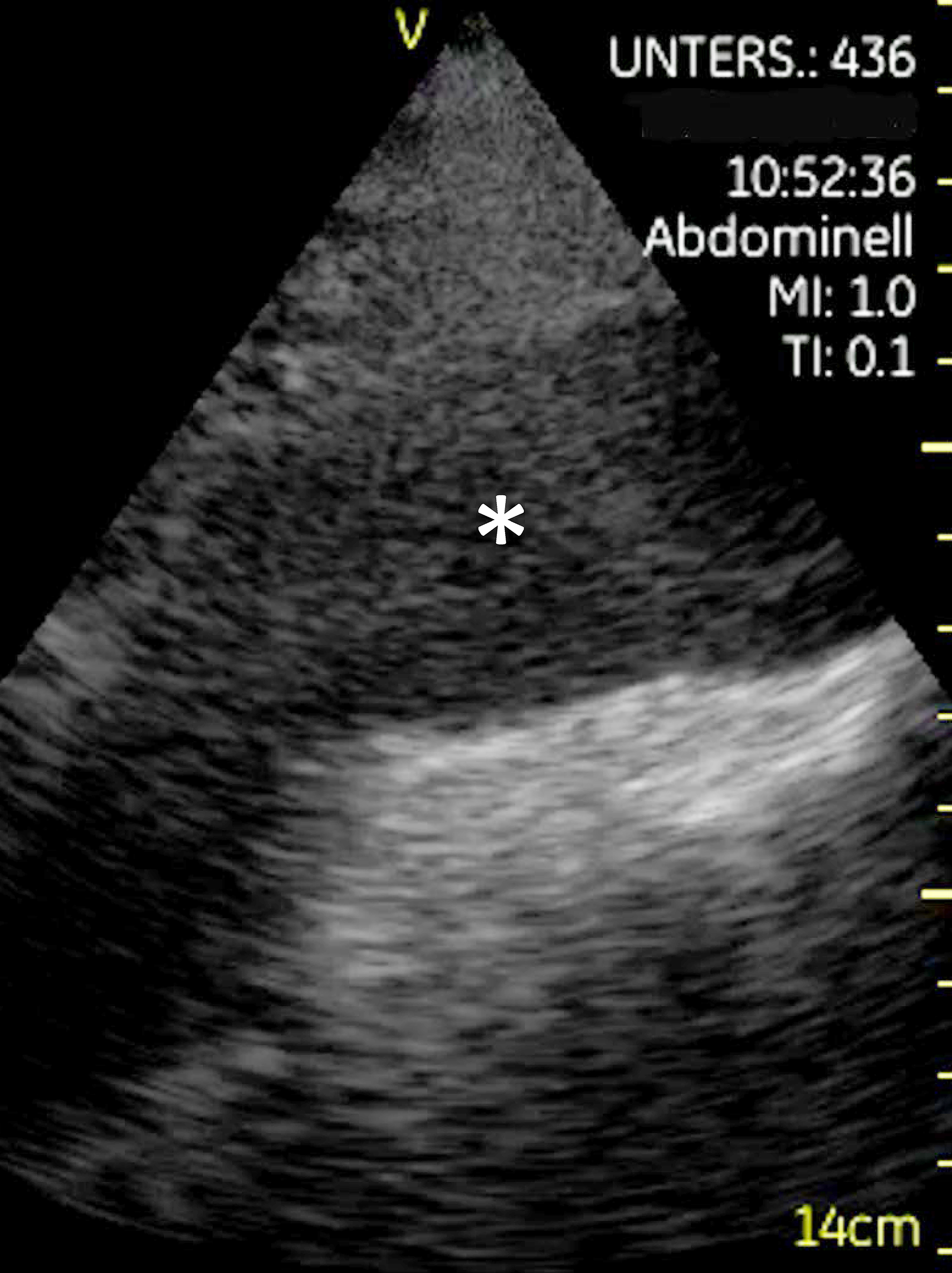

Supplement: Supplementary file 6 — Online Resource 5 a and b Abdominal aorta with aneurysm (*) (TIF 12326 KB) [file 345_2022_4018_MOESM6_ESM.tif]

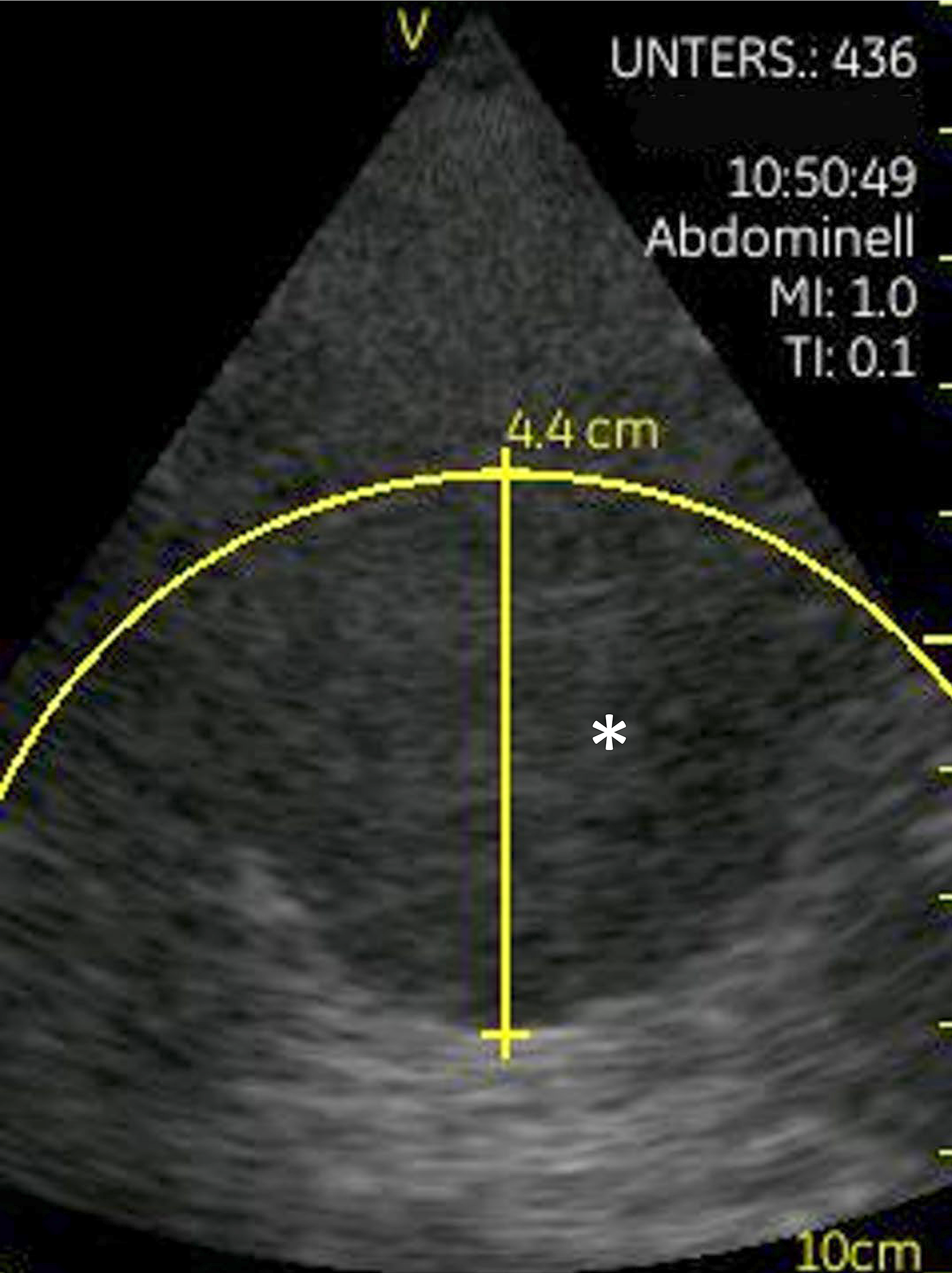

Supplement: Supplementary file 7 — Supplementary file7 (TIF 12330 KB) [file 345_2022_4018_MOESM7_ESM.tif]

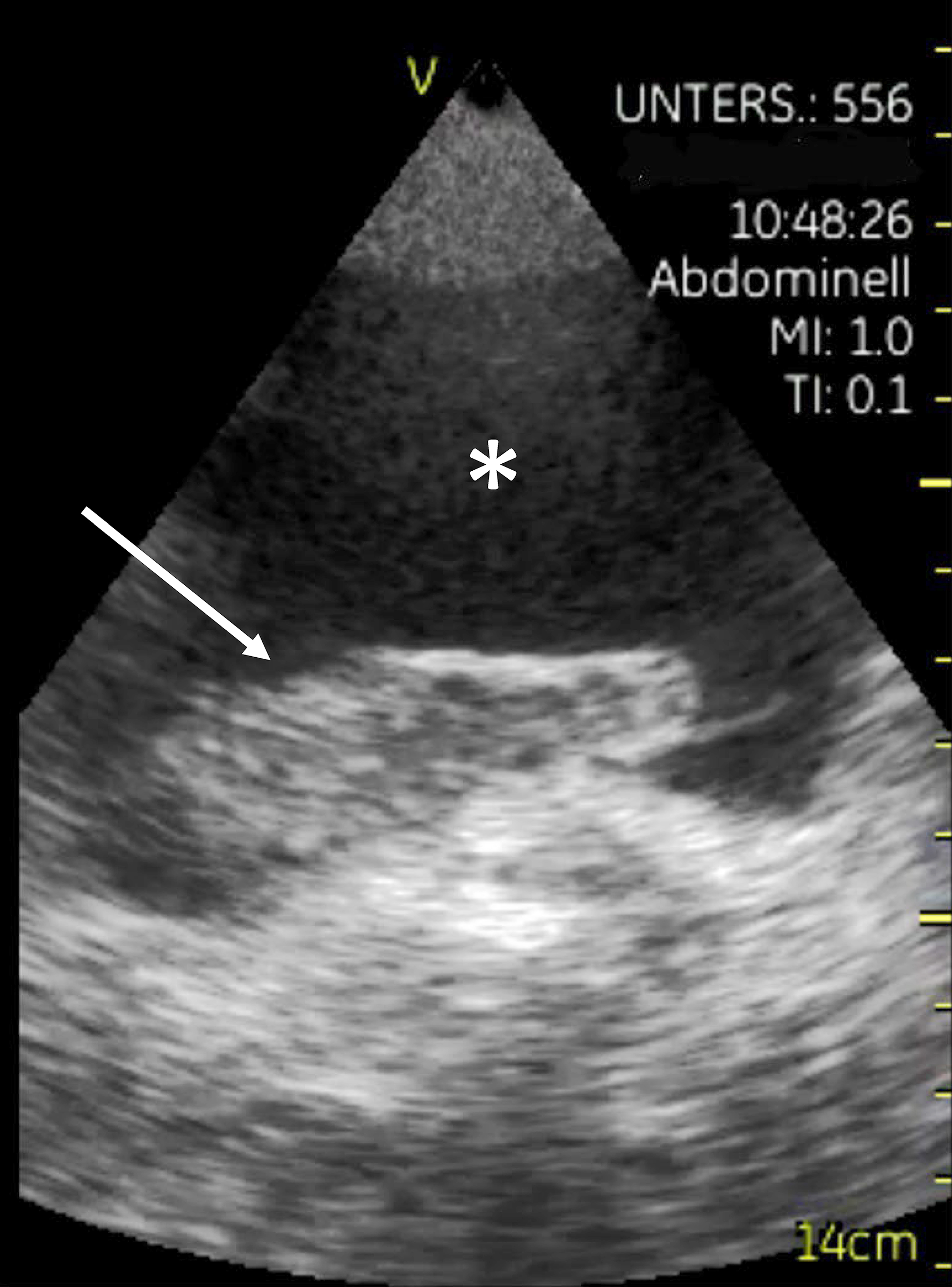

Supplement: Supplementary file 8 — Online Resource 6 Blood clot (→) in the urinary bladder (*) (TIF 12460 KB) [file 345_2022_4018_MOESM8_ESM.tif]

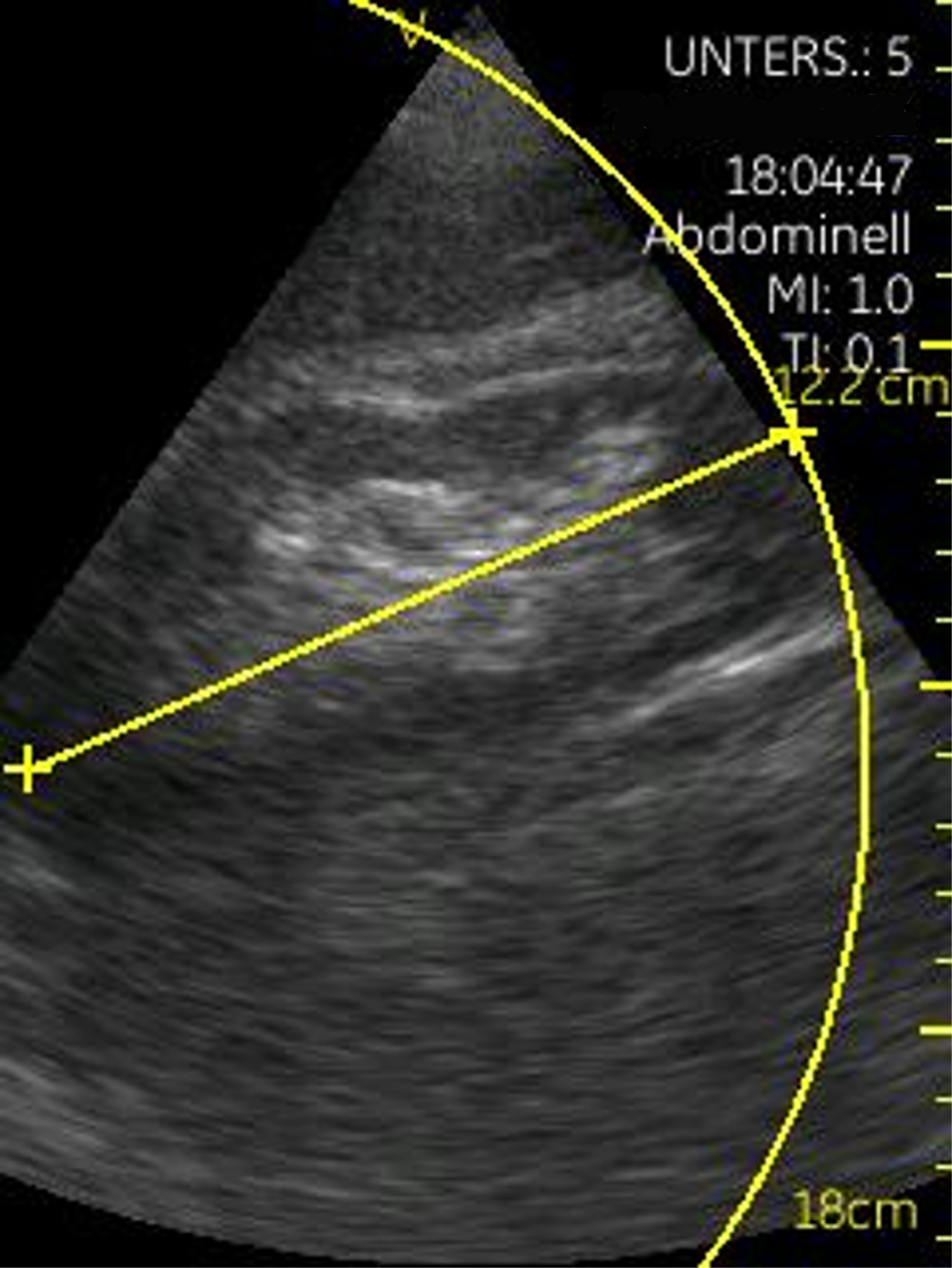

Supplement: Supplementary file 9 — Online Resource 7 Kidney: measurement of the renal size (TIF 2699 KB) [file 345_2022_4018_MOESM9_ESM.tif]

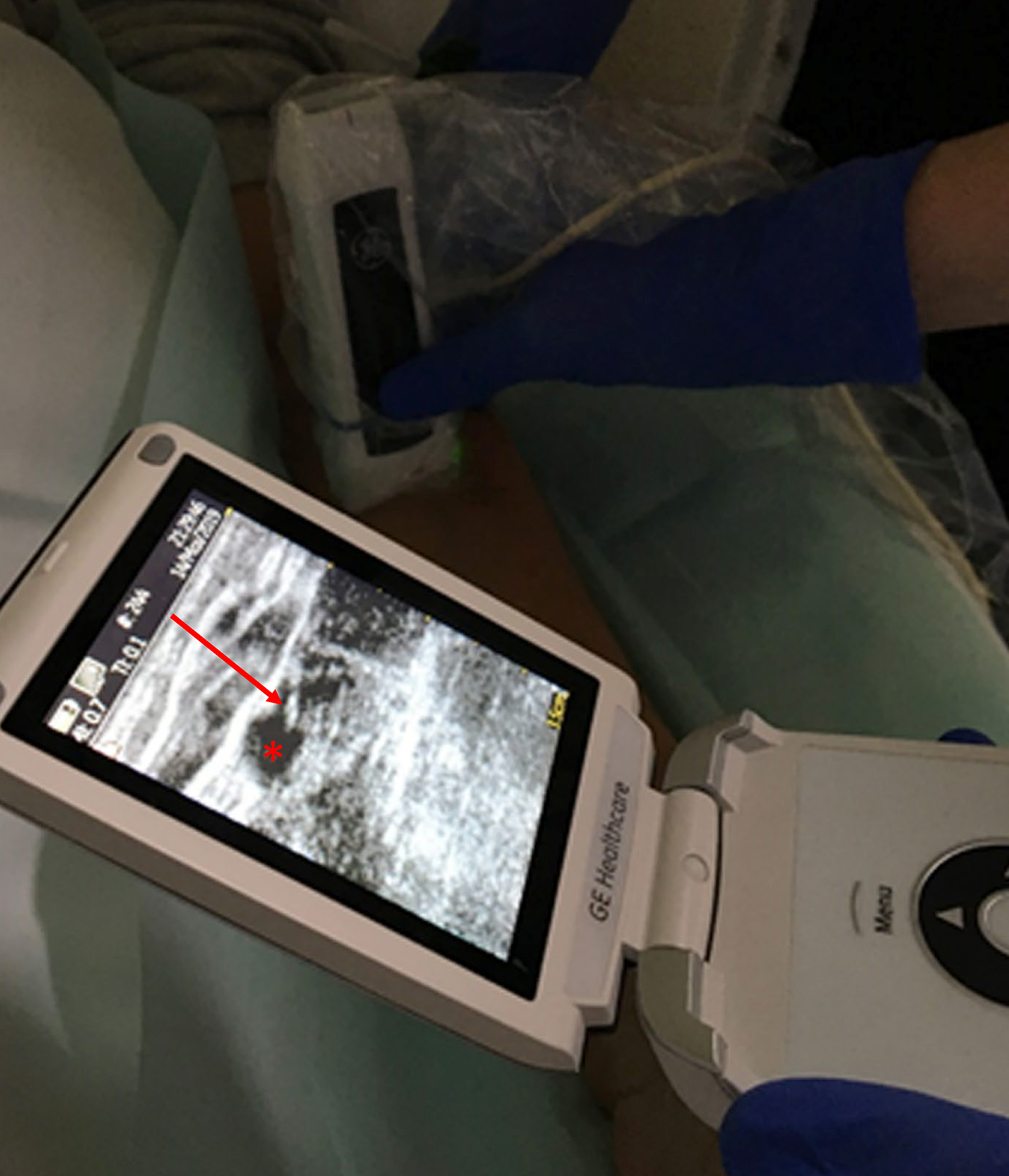

Supplement: Supplementary file 10 — Online Resource 8 Cannulation of a haemodialysis fistula: on the monitor of the HHUD you can see the needle (→) placed in the haemodialysis fistula (*) (TIF 10746 KB) [file 345_2022_4018_MOESM10_ESM.tif]
